# Survival, Retention, and Selective Proliferation of Lymphocytes Is Mediated by Gingival Fibroblasts

**DOI:** 10.3389/fimmu.2018.01725

**Published:** 2018-07-25

**Authors:** Carolyn G. J. Moonen, Sven T. Alders, Hetty J. Bontkes, Ton Schoenmaker, Elena A. Nicu, Bruno G. Loos, Teun J. de Vries

**Affiliations:** ^1^Department of Periodontology, Academic Center for Dentistry Amsterdam (ACTA), University of Amsterdam and Vrije Universiteit, Amsterdam, Netherlands; ^2^Department of Oral Cell Biology, Academic Center for Dentistry Amsterdam (ACTA), University of Amsterdam and Vrije Universiteit, Amsterdam, Netherlands; ^3^Department of Clinical Chemistry, Medical Immunology, Vrije Universiteit Medical Center (VUMC), Amsterdam, Netherlands; ^4^Opris Dent SRL, Sibiu, Romania

**Keywords:** osteoclast formation, osteoimmunology, gingival fibroblast, peripheral blood cells, cell–cell interaction, leukocytes

## Abstract

Periodontitis, a chronic inflammatory disease of the periodontium, is characterized by osteoclast-mediated alveolar bone destruction. Gingival fibroblasts (GFs) present in the bone-lining mucosa have the capacity to activate the formation of osteoclasts, but little is known about which local immune cells (co-)mediate this process. The aim of this study was to investigate the cellular interactions of GFs with immune cells, including the contribution of GFs to osteoclast formation and their possible role in the proliferation of these immune cells. In addition, we investigated the expression of adhesion molecules and the inflammatory cytokines that are evoked by this interaction. GFs were cocultured with peripheral blood mononuclear cells (PBMCs), CD14+ monocytes or peripheral blood lymphocytes (PBLs) for 7, 14, and 21 days. After 21 days, comparable numbers of multinucleated cells (osteoclasts) were found in gingival fibroblast (GF)-PBMC and GF-monocyte cocultures. No osteoclasts were formed in GF-PBL cocultures, indicating that the PBLs present in GF-PBMC cocultures do not contribute to osteoclastogenesis. Persisting mononuclear cells were interacting with osteoclasts in GF-PBMC cocultures. Remarkably, a predominance of CD3+ T cells was immunohistochemically detected in GF cocultures with PBLs and PBMCs for 21 days that frequently interacted with osteoclasts. Significantly more T, B (CD19+), and NK (CD56+CD3−) cells were identified with multicolor flow cytometry in both GF-PBMC and GF-PBL cocultures compared to monocultures without GFs at all time points. GFs retained PBLs independently of the presence of monocytes or osteoclasts over time, showing a stable population of T, B, and NK cells between 7 and 21 days. T helper and cytotoxic T cell subsets remained stable over time in GF cocultures, while the number of Th17 cells fluctuated. Lymphocyte retention is likely mediated by lymphocyte-function-associated antigen-1 (LFA-1) expression, which was significantly higher in GF-PBL cultures compared to GF-monocyte cultures. When assessing inflammatory cytokine expression, high tumor necrosis alpha expression was only observed in the GF-PBMC cultures, indicating that this tripartite presence of GFs, monocytes, and lymphocytes is required for such an induction. Carboxyfluorescein succinimidyl ester-labeling showed that only the CD3+ cells proliferated in presence of GFs. This study demonstrates a novel role for GFs in the survival, retention, and selective proliferation of lymphocytes.

## Introduction

Periodontitis is a multifactorial chronic inflammatory disease affecting the supporting tissues of the teeth, the periodontium. In periodontal health, a tolerant host inflammatory immune response is thought to protect the host against periodontopathogens, resulting in an immune response with a resolving character (1). On the other hand, in patients with periodontitis, the inflammatory immune response is chronic and persists, resulting in an overproduction and stimulation of osteoclasts leading to bone resorption and potentially tooth loss (2, 3). It is generally accepted that periodontitis is initiated by microbial products such as lipopolysaccharide (4, 5). These virulence factors are recognized by pattern-recognition receptors and initiate a local inflammatory immune response. Immune cells, including neutrophils and other leukocytes, which circulate in the blood are recruited to the inflamed periodontium to mediate an effective response (6, 7). They extravasate from the blood vessels and invade the soft tissue of the periodontium, where they likely interact with fibroblasts. At the site of inflammation, cytokines and proteolytic enzymes are produced by immune cells such as neutrophils, T cells (CD3+), B cells (CD19+/CD20+), NK cells (CD56+CD3−), monocytes (CD14+), and macrophages, but also fibroblasts (5, 8–10). They contribute to the local activation of inflammatory cells and the increased formation of bone-degrading tartrate resistant acid phosphatase (TRACP) positive multinucleated osteoclasts (10–12).

Gingival fibroblasts (GFs), present in the alveolar bone-lining mucosa (i.e., the gingiva), are thought to play a role in the recruitment of immune cells toward the inflamed periodontium. These fibroblasts may contribute to the progression of periodontitis by controlling the balance of osteoclastogenesis and osteoblastogenesis by which an altered balance can lead to pathological alveolar bone destruction (8–10). There is extensive literature suggesting that monocytes can differentiate into pre-osteoclasts and eventually fuse into multinucleated bone-resorbing osteoclasts (13, 14). The role of GFs in osteoclastogenesis was shown by *in vitro* cocultures of GFs with peripheral blood mononuclear cells (PBMCs), where osteoclast-like cells formed after 21 days (15). Cell–cell contact between gingival or periodontal ligament (PDL) fibroblasts from the periodontium and osteoclast precursors is required for osteoclastogenesis since hardly any osteoclasts form without coculturing with any of these two types of fibroblasts (15–17). Thus, these cellular interactions are important in the survival of osteoclast precursors where fibroblasts apparently provide the appropriate signals. While osteoclasts are induced by GFs in fibroblast-PBMC cocultures, it is unknown which other mononuclear cell types from the PBMCs persist throughout this differentiation and whether these cells also play a role in osteoclastogenesis while cocultured with GFs. We hypothesized that GFs play a role in the retention, survival, and proliferation of lymphocytes. In order to study this, we investigated the role of GFs in cellular interactions with the leukocyte subsets present in cocultures of PBMCs, peripheral blood lymphocytes (PBLs), and isolated monocytes.

## Materials and Methods

### Gingival Fibroblasts

Gingival fibroblasts were previously isolated (15, 18) from discarded third molars (wisdom teeth) from 18 healthy individuals and stored in liquid nitrogen. Sampling from the donors was conducted at the Academic Center for Dentistry Amsterdam (ACTA), the Netherlands. Informed consent was obtained from all individuals and samples were coded to guarantee the anonymity of the donors as required by Dutch law. Researchers handling the fibroblasts (Carolyn G. J. Moonen, Sven T. Alders, Ton Schoenmaker, and Teun J. de Vries) could not retrieve the identity of the donors. For the current study, GFs (passages 4–6) were individually recovered in culture medium (Dulbecco’s minimal essential medium, Gibco BRL, Paisley, Scotland) supplemented with 10% fetal calf serum (FCS, Hyclone, Logan, USA), and 1% antibiotics [100 U/mL penicillin, 100 µg/mL streptomycin, and 250 ng/mL amphotericin B (Antibiotic antimycotic solution, Sigma Aldrich, St. Louis, MO, USA)], and cultured in a humidified atmosphere of 5% CO_2_ in air at 37°C. All GFs were used as individual entities, e.g., not mixed with GFs of other donors.

### Blood Cell Isolation for Osteoclastogenesis

Peripheral blood mononuclear cells were isolated from buffy coats (Sanquin, Amsterdam, The Netherlands) of healthy donors by standard density gradient centrifugation with Ficoll-Paque. First, buffy coats were diluted 1:1 in 1% PBS-citrate (pH 7.4) and subsequently 25 mL of the diluted buffy coat was carefully layered on 15 mL Lymphoprep (Axis-Shield Po CAS, Oslo, Norway) and centrifuged for 30 min at 800 RCF without brake. The interphase, containing PBMCs, was washed thrice in 1% PBS-citrate and recovered in culture medium. From the PBMCs, monocytes were isolated after incubation for 15 min on ice with saturating concentrations of biotinylated CD14-conjugated magnetic microbeads (Miltenyi Biotec, Bergisch Gladbach, Germany). The cell-bead suspension was centrifuged at 400 RCF for 10 min and washed in PBS containing 0.5% bovine serum albumin (BSA) and 2 mM Tris–EDTA to remove unbound magnetic antibodies (Abs). The cell-bead suspension was transferred onto a column containing a ferromagnetic sphere matrix and placed in the magnetic field of the magnetic-assisted cell sorter (MACS). PBLs were collected as the unlabeled, CD14 negative fraction, while the microbead-labeled (CD14 positive) monocytes were eluted and collected after removal of the column from the MACS. Finally, cells were recovered in culture medium. To evaluate purity, the positively enriched cell fraction was tested with efluor-450 labeled anti-human CD14 (Table 1) and found to be >96% CD14+ monocytes as confirmed by flow cytometry (FACSverse™, BD Biosciences, Piscataway, USA). Both the CD14-positive (>96% CD14+) and negative (<0.9% CD14+) fractions were used for experiments and referred to as monocytes and PBLs, respectively.

**Table 1:** Reagent list used for flow cytometry experiments.

Instrument: BD biosciences FACSVerse™					
Antibody	Fluorochrome	Vendor/cat no./clone	Dilution factor	Laser line (nm)	Emission filters
Anti-human CD56	PE	eBiosciences by ThermoFisher Scientific/#12056742/MSSB	50	488	586/42
Anti-human CD3	BV510	eBiosciences by ThermoFisher Scientific/#563109/UCHT1	50	405	528/45
Anti-human CD19	APC	eBiosciences by ThermoFisher Scientific/#17019842/SJ25C1	100	640	660/10
Anti-human CD14	Efluor 450	BD biosciences/#48014942/61D3	50	405	448/45
Anti-human CD4	PerCP-Cy™5.5	eBiosciences by ThermoFisher Scientific/#45004942/RPA-T4	100	488	700/54
Anti-human CD8	APC-Cy™780	eBiosciences by ThermoFisher Scientific/#47008742/SK1	100	640	783/56
Anti-human CD196/CCR6	PE-Cy™7	eBiosciences by ThermoFisher Scientific/#25196941/R6H1	50	488	783/56

### Osteoclastogenesis

Gingival fibroblasts (1.5 × 10^4^ per well, *n* = 4) were seeded in duplicate and allowed to attach overnight in 48-well plates in triplicate. Monocytes (1 × 10^5^ per well), PBL (4 × 10^5^ per well), or PBMCs (5 × 10^5^ per well) were seeded on top of the GFs. Concentrations were chosen based on the physiological ratio of monocyte:PBL in blood *in vivo* of 1:4. Cultures were refreshed every 3–4 days and maintained for 21 days at 37°C in a humidified 5% CO_2_ in air incubator. Monocultures of PBMCs, PBL, and monocyte fractions were cultured as controls.

### TRACP Staining

After 21 days, cells were fixed in 4% PBS-buffered formaldehyde for 10 min and washed in PBS. Multinucleated cells (MNCs) were identified by enzyme-histochemistry with a TRACP staining (Sigma-Aldrich) according to the manufacturer’s protocol. Nuclei were counterstained with 4′,6-diamidino-2-fenylindool (DAPI) for 5 min. TRACP + MNCs were counted using a combination of light and fluorescence microscopy (Leica DFC320; Leica Microsystems, Wetzlar, Germany) and considered to be osteoclasts when TRACP positive with at least three nuclei. Five standardized pictures per well were analyzed at a magnification of 20× for the number of MNCs containing at least three nuclei and expressed as MNCs/well. TRACP + MNCs were counted per well and categorized into groups of 3–5, 6–10, or 11–20 nuclei per cell. For four independent experiments with four different buffy coats and seven different gingival fibroblast (GF) sources, the average of each well of duplicates was used for analysis.

### Immunohistochemical Staining of CD3+ T and CD20+ B Cells

An immunohistochemical staining for CD3+ or CD20+ cells for the detection of interacting mononuclear TRACP negative cells was performed after 7, 14, and 21 days of PBMC cultures with or without GFs. Cells were washed before and after fixation with 4% PBS-buffered formaldehyde for 10 min. To block background staining of the secondary antibody, the fixed cells were incubated with 10% normal goat serum, 1% BSA, and 0.05% Triton X100 for 1 h. For identification of T and B cells, anti-CD3 (BD Biosciences, Piscataway, USA) or anti-CD20 (Dako, Santa Clara, CA, USA), respectively, was added and incubated for 1 h at 4°C in the dark. As isotype control, IgG2a antibody was used and showed no staining throughout the experiments. After washing three times with PBS, the secondary antibody goat anti-rat Alexa 488 (2 mg/mL Invitrogen, Eugene, OR, USA), 400 × diluted in culture medium, was added to visualize antibody binding and incubated for 1 h at 4°C in the dark. To stain nuclei, cells were washed with PBS before and after staining with DAPI (200 × diluted in PBS) for 5 min [room temperature (RT), dark]. For assessing the number and percentage of CD3+ and CD20+ cells, all nuclei were counted in two standardized pictures per well using a fluorescent light microscope (Leica DFC320). Large nuclei could be attributed to GFs and were excluded. Upon switching to the appropriate light filter, the number of CD3+ and CD20+ (green) cells was subsequently counted. The proportions of T and B cells were calculated by dividing the numbers of CD3+ and CD20+ cells, respectively, by the total number of mononuclear cells per well. The difference in the proportion of CD3+ and CD20+ leukocytes between the coculture and monoculture was further analyzed. The average of each well was used for analysis.

### Heterogeneous Cell Population Characterization With Flow Cytometry

For confirmation of immunohistochemical CD3+ T and CD20+ B cell identification, heterogeneous cell suspensions of two independent experiments (*n* = 2 buffy coats, *n* = 10 GFs; five per experiment) were characterized over time using flow cytometry. Various cell types were identified: CD3+ (T cells), CD4+ [T helper (Th) cells], CD8+ [cytotoxic T (Tc) cells], CD196+/CCR6 (Th17 cells), CD19+ (B cells), CD56+CD3− (NK cells), and CD14+ (monocytes). As controls, isolated cell suspensions (PBMCs, monocytes, and PBLs) were characterized before coculturing with GFs for one experiment. After 7, 14, and 21 days of culture, cells were trypsinized with 0.05% Trypsin in 0.5 mM EDTA in PBS for 15 min at 37°C, duplicates were pooled, washed, and suspended in FACS buffer (20 µg/mL NaN_3_, 0.5% BSA in PBS). Then, the cell suspension was incubated (4°C, dark) with a mixture of monoclonal antibodies (mAb) presented in Table 1.

**Figure 1:**
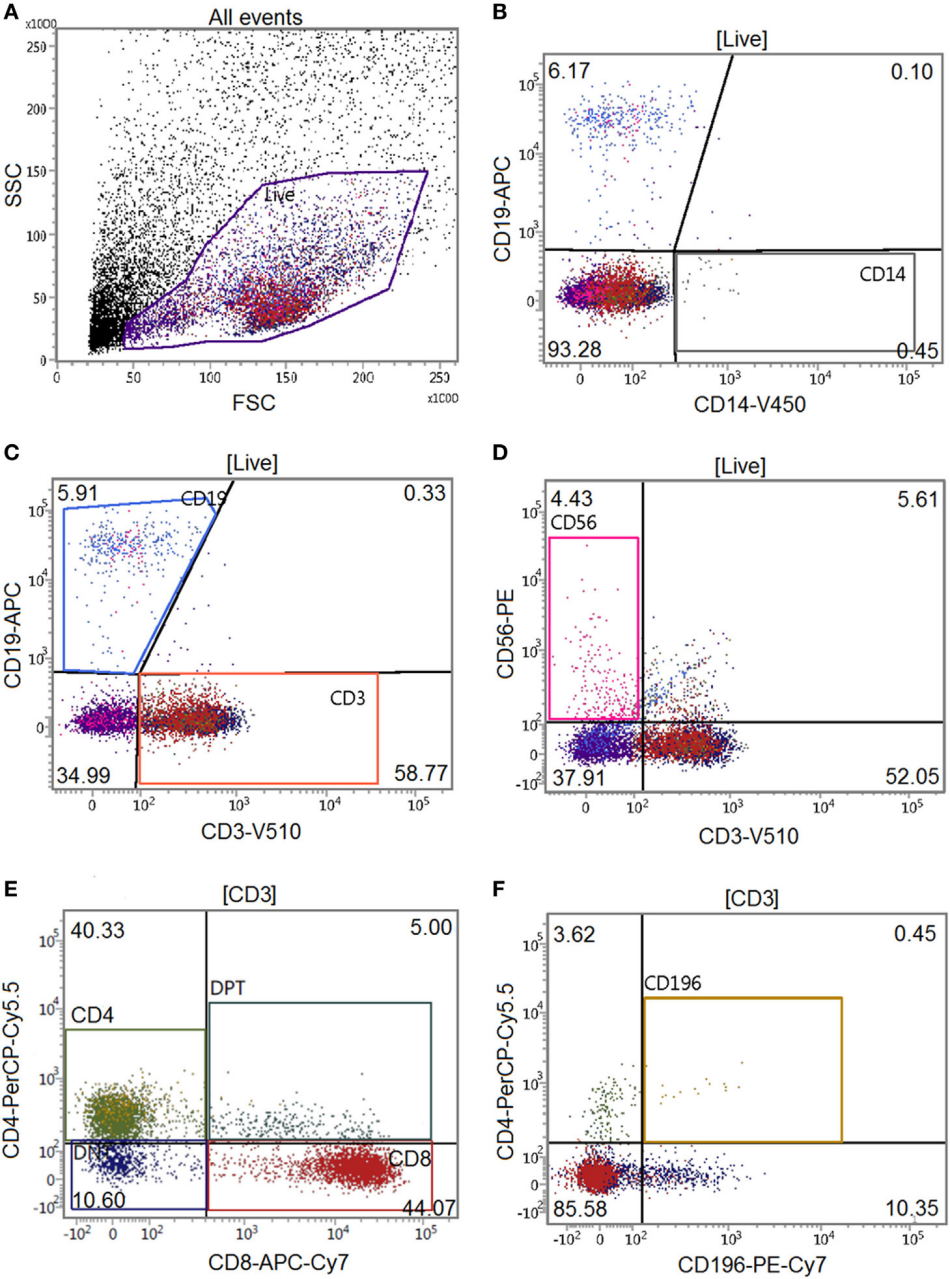
Gating tree of flow cytometry experiments. The antibody combination for flow cytometric experiments was CD14-V450/CD19-APC/CD3-V500/CD56-PE/CD4-PerCPCy5.5/CD8-APC-Cy7/CD196-PE-Cy7 (Table 1). Acquisition and analysis were performed on a BD FACSverse flow cytometer using associated FACSuite software. The number in quadrants indicate percentages of cells in the corresponding gating. The gating tree was set as follows: **(A)** forward scatter/sideward scatter represents the distribution of cells in the light scatter based on size and intracellular composition, respectively, where the live population is encircled in purple. In the “live” population, **(B)** CD14+, **(C)** CD19+, CD3+, **(D)** CD56+CD3− cells were identified. In the CD3+ gating, **(E)** CD4+, CD8+, and **(F)** CD196+ populations were identified. Here, a characterization of a gingival fibroblast-peripheral blood mononuclear cell coculture after 21 days is shown.

Optimal antibody concentrations and spillover values were established during previously performed titration experiments. Multicolor compensation was performed with PBMCs, and these settings were applied during all following experiments. After 30 min, the cell-mAb mixture was washed with FACS buffer to remove unbound antibodies and samples were kept on ice and in the dark until further analysis. Flow cytometric analysis was performed on a BD FACSverse™ flow cytometer (BD Biosciences) with a medium flow rate (63 µL/min) where at least 10,000 cells were analyzed per sample. Quantification of cells was performed by automatic volumetric measurements over the entire acquisition time per sample. Gating of the live population was based on PBMC controls without GFs to exclude GF cells in coculture data (Figure 1). Flow data were analyzed using FACSuite™ software (v1.0.5, BD Biosciences).

### Gene Expression Analysis

After 14 and 21 days of culturing, RNA was extracted from the GF (*n* = 6) mono- and cocultures with PBMCs, PBLs, and monocyte fractions using a commercial spin-column kit (RNeasy Mini kit, Qiagen, Düsseldorf, Germany) according to the manufacturer’s protocol. Total isolated RNA concentrations were spectrophotometrically (Synergy, Biotek, Winooski, VT, USA) quantified. Reverse transcription was performed using an MBI Fermentas cDNA synthesis kit (Thermofischer Scientific) and real-time quantitative PCR (qPCR) was performed with a Roche Lightcycler 480 II. The primers used for analysis are listed in Table 2. Relative gene expression was normalized by the geometric mean expression of the housekeeping genes porphobilinogen deaminase (PBGD) and hypoxanthine phosphoribosyltransferase (HPRT) following the comparative cycle threshold (Ct) method and presented as mean relative fold expression (2^−Δct^) ± SEM.

**Table 2:** Primer sequences used for quantitative PCR experiments.

Gene		5′ → 3′ sequence
ICAM-1	Forward	5′ TGAGCAATGTGCAAGAAGATAGC 3′
	Reverse	5′ CCCGTTCTGGAGTCCAGTACA 3′

IL-1β	Forward	5′ CTTTGAAGCTGATGGCCCTAAA 3′
	Reverse	5′ AGTGGTGGTCGGAGATTCGT 3′

LFA-1	Forward	5′ GAGCTGGTGGGAGAGATCGA 3′
	Reverse	5′ GAGGCGTTGCTGCCATAGA 3′

PBGD	Forward	5′ TGCAGTTTGAAATCATTGCTATGTC 3′
	Reverse	5′ AACAGGCTTTTCTCTCCAATCTTAGA 3′

TNFα	Forward	5′ CCCAGGGACCTCTCTCTAATCA 3′
	Reverse	5′ GCTTGAGGGTTTGCTACAACATG 3′

VCAM	Forward	5′ ACAAAGTTGGCTCACAATTAAGAAGTT 3′
	Reverse	5′ TGCAAAATAGAGCACGAGAAGCT 3′

VLA-4	Forward	5′ CGAACCGATGGCTCCTAGTG 3′
	Reverse	5′ CACGTCTGGCCGGGATT 3′

### Cytokine Production

Conditioned media was taken from the GF (*n* = 5) mono- and cocultures with PBMCs, PBLs, and monocyte fractions at 7, 14, and 21 days. Concentrations of interleukin-1beta (IL-1β) and tumor necrosis factor alpha (TNF-α) were measured with enzyme-linked immunosorbent assays (ELISA, R&D systems, Minneapolis, MN, USA) according to the manufacturer’s instructions.

### Proliferation Assay

Before coculturing, PBMC and PBL cells were labeled with Celltrace^TM^ carboxyfluorescein succinimidyl ester (CFSE, Invitrogen by ThermoFischer, Eugene, OR, USA) for the detection of cell proliferation. PBMCs (10 × 10^6^ cells/mL) were washed in 5% FCS in PBS and incubated at 37°C with 3 µM CFSE. After 7 min incubation, cells were washed for 15 min at RT in the dark and washed again in 5% FCS in PBS. Then, the cell pellet was suspended in culture medium and checked for labeling efficiency with flow cytometry. Finally, CFSE-labeled cells were cocultured with GFs for 7, 14, and 21 days in the dark. Proliferation by means of a decrease in CFSE signal detected as a novel cell population that had distinctly lower CFSE signal was detected with flow cytometry (FITC channel: excitation 492 nm, emission 517 nm) for live, CD3+, CD8+ gated cells. Quantification of proliferation was expressed as a percentage of CD3+CD8−CD4+ or CD3+CD8+ cells. Shifts of CFSE label were also analyzed for CD19+, CD56+, and CD14+ cells, but a demarcated population was not visible for these cell types.

### Statistics

All data were analyzed using GraphPad Prism software (version 6.07, La Jolla, CA, USA). Data are calculated and presented as means ± SEM. Normality of data distribution was assessed with the D’agostino-Pearson test. Normally distributed data were compared with parametric paired *T*-tests for osteoclast, T and B cell quantifications, relative gene expression, cytokine production, absolute cell counts, and CFSE data from flow cytometry analyses. For non-normally distributed data, percentages of various cell subtypes from flow cytometry experiments were statistically compared using the Kruskal–Wallis test. Differences were considered significant at *p* < 0.05.

## Results

### GFs Enhance Osteoclastogenesis

**Figure 2:**
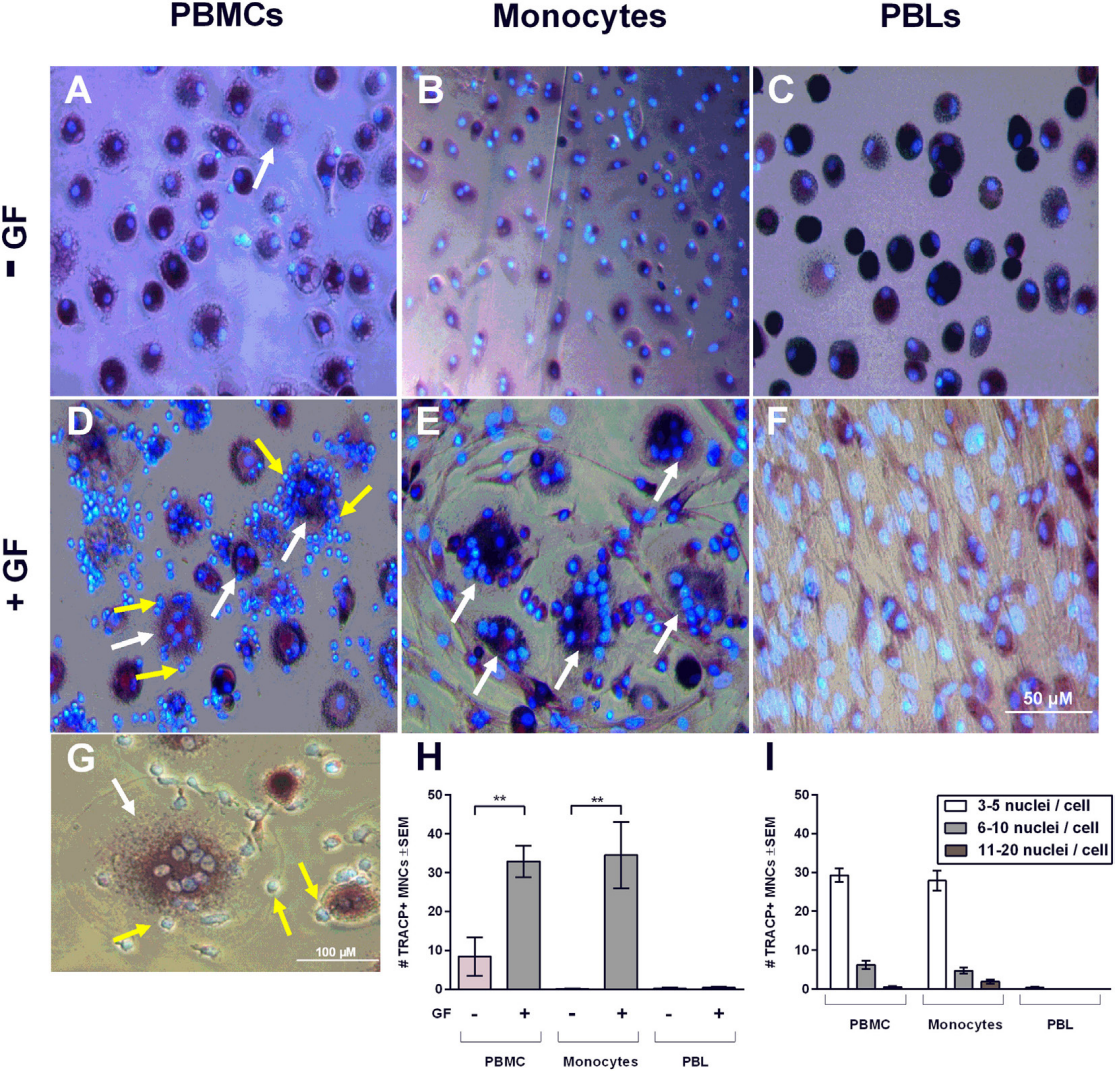
Gingival fibroblasts (GFs) enhance osteoclastogenesis in the presence of monocytes. Micrographs of monocultures of **(A)** peripheral blood mononuclear cell (PBMCs), **(B)** monocytes, and **(C)** peripheral blood lymphocytes (PBLs) after 21 days. Micrographs of cocultures of GFs with **(D)** PBMCs, **(E)** monocytes, and **(F)** PBLs after 21 days of culture. Cells were stained for TRACP activity (purple) and nuclei were stained with 4′,6-diamidino-2-fenylindool (blue). TRACP+ multinucleated cells (MNCs) were considered to be osteoclasts when TRACP+ with at least three nuclei. Osteoclasts are depicted with white arrows and are exclusively visible in conditions with cocultures of GFs with PBMCs and monocytes **(D,E)**. All micrographs are representatives for four independent experiments with seven different GF sources. Scale bar represents 50 µM. **(G)** Osteoclast (white arrow) present in coculture of GFs with PBMCs. Interacting TRACP-negative mononuclear cells with osteoclasts are depicted with yellow arrows **(D,G)**. Scale bar represents 100 µM. **(H)** Quantification of the number of TRACP+ MNCs per five standardized pictures per well in monocultures and cocultures of PBMCs, monocytes, or PBLs after 21 days. Significantly more osteoclasts were found in cocultures of GFs with PBMCs or monocytes when compared to monocultures, *n* = 4 GF donors, **p < 0.01. **(I)** Number of nuclei per TRACP+ cell in GF cocultures with PBMCs, monocytes, and PBLs after 21 days. Mainly, osteoclasts with 3–5 nuclei were found in cocultures with monocytes.

Peripheral blood mononuclear cells, monocytes, and PBLs were either monocultured or cocultured with GFs for 21 days and stained with TRACP and DAPI to microscopically visualize TRACP + MNCs (considered to be osteoclasts). These cells were observed in all conditions without GFs (Figures 2A–C), even in PBL. Judging from the size and form, these TRACP+ cells probably arose from contaminating monocytes. In line with previous reports, osteoclasts only formed in cocultures that contained monocytes (i.e., the PBMC and the isolated monocyte cultures). In the presence of GFs and monocytes, distinctly more osteoclasts were observed than in monocultures (Figures 2D,H). The presence of GFs significantly increased the number of osteoclasts that were formed after 21 days (*p* = 0.008 for PBMC cocultures, *p* = 0.007 for monocyte cocultures, Figure 2H). However, no significant differences (*p* = 0.87) were observed between PBMC and monocyte cocultures. This confirmed that monocytes are the main osteoclast precursors in the PBMC fraction and are thereby the crucial cell fraction of PBMCs for osteoclast formation. PBMCs, monocytes, and PBLs hardly survived on plastic without GFs after 21 days of culture in culture media (Figure 2H), indicating that GFs improve the survival of pre-osteoclasts and PBLs and clearly enhance osteoclastogenesis *in vitro*. Interestingly, GFs retract in cultures where osteoclasts are formed (Figures 2D,E), whereas no migration of GFs is seen in the absence of monocytes (Figure 2F). Monocultures of GFs have a similar appearance as shown in Figure 2F (data not shown). During osteoclastogenesis in the presence of PBMCs, persisting mononuclear and TRACP-negative cells are present around osteoclasts (yellow arrows, Figures 2D,G) as well as adhered to GF. These persistent TRACP-negative mononuclear cells were not visible in cocultures of monocytes (Figure 2E). Accordingly, interacting mononuclear cells in monocyte GF cocultures (Figure 2E) were TRACP+, which indicates that these are adherent, possibly fusing monocytes. Also, the interacting TRACP-negative cells were smaller than the TRACP+ mononuclear cells in GF-monocyte cocultures (compare Figures 2D,E). Collectively, this suggests that the interacting cells in PBMC cocultures are PBLs. Finally, the number of nuclei per osteoclast was assessed (Figure 2I), reflecting the size of the osteoclast (19). The majority of osteoclasts were of small size (3–5 nuclei per cell), both in GF-PBMC and in GF-monocyte cocultures (Figure 2I, white bars).

### Immunohistochemical Identification of Persisting Mononuclear Cells in Cocultures

**Figure 3:**
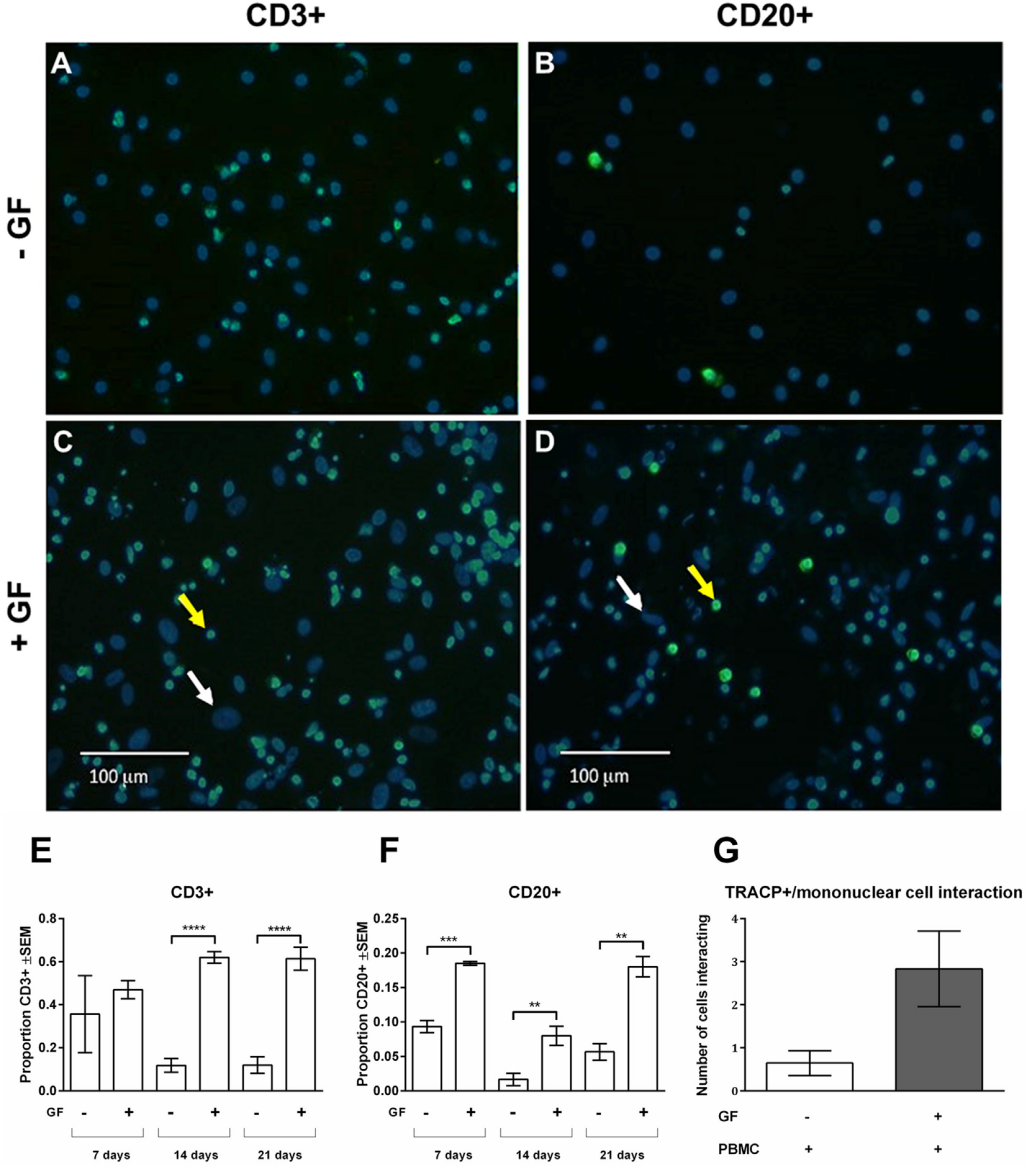
Gingival fibroblasts (GFs) are involved in the retention and survival of T and B cells after coculture with peripheral blood mononuclear cells (PBMCs). Overlay of immunohistochemical stainings with 4′,6-diamidino-2-fenylindool (DAPI) and CD3+ or CD20+ staining of **(A,B)** monocultures of PBMCs and **(C,D)** GF cocultures with PBMCs after 21 days of culture. **(A,C)** CD3+ and **(B,D)** CD20+ stainings (green) were performed to identify T and B cells, respectively. Nuclei are stained with DAPI (blue). GF nuclei are depicted with white arrows. CD3+ cells **(A,C)** and CD20+ cells **(B,D)** are depicted with yellow arrows, which are abundantly present in cocultures with GFs. Micrographs are representatives for two independent experiments with five different GF sources. Scale bars represent 100 µM. Proportions of CD3+ **(E)** and CD20+ **(F)** cells of total mononuclear cells after 7, 14, and 21 days were quantified from two standardized pictures per well. Significantly more CD3+ and CD20+ cells were identified in GF cocultures. The number of mononuclear cells interacting with TRACP+ cells **(G)** in PBMC monoculture (white bar) and GF coculture (gray bar). No significant difference (*p* = 0.0561) between mono- and cocultures was found. *n* = 5 GF donors, *n* = 2 buffy coats **p < 0.01, ***<0.001, ****<0.0001.

As shown in Figures 2D,G, persisting mononuclear TRACP-negative cells interacting with osteoclasts and GFs were observed in PBMC cocultures. Subsequently, persisting mononuclear cells were immunohistochemically identified with T and B cell markers (anti-CD3+ and anti-CD20+, respectively) (Figure 3). Figure 3 depicts overlays of T (Figures 3A,C) and B cell (Figures 3B,D) stainings shown in green, and DAPI staining in blue of PBMC monocultures (Figures 3A,B) and PBMC-GF cocultures (Figures 3C,D). Proportions of T or B cells were calculated by the number of CD3+ or CD20+ cells (yellow arrows, Figures 3C,D) divided by the number of DAPI+ mononuclear nuclei, excluding large GF nuclei (white arrows, Figures 3C,D). More CD3+ and CD20+ cells were observed in the presence of GFs (Figures 3C,D, respectively). Subsequently, quantification of immunohistochemical stainings demonstrated a significantly higher proportion of CD3+ and CD20+ cells after 21 days of coculture. The proportion of CD3+ (Figure 3E) was significantly higher in cocultures after 14 days (*p* < 0.0001), than what was observed after 7 days for B cells (Figure 3F). After 14 days, the proportion of B cells decreases and recovers after 21 days. Furthermore, the number of leukocytes interacting with TRACP+ cells was counted (Figure 3G). A tendency toward more interacting cells in the GF cocultures compared to PBMC monocultures was observed; however, this did not reach statistical significance, *p* = 0.0561. This indicates that the presence of GF does not increase the interactions between TRACP+ cells with T or B cells. In monocultures of GFs, no CD3+ or CD20+ staining was observed throughout experiments. Additionally, negative controls (IgG2a isotype staining) showed an absence of staining throughout the experiments.

### PBL Cells Survive for at Least 21 Days in Presence of GFs

**Figure 4:**
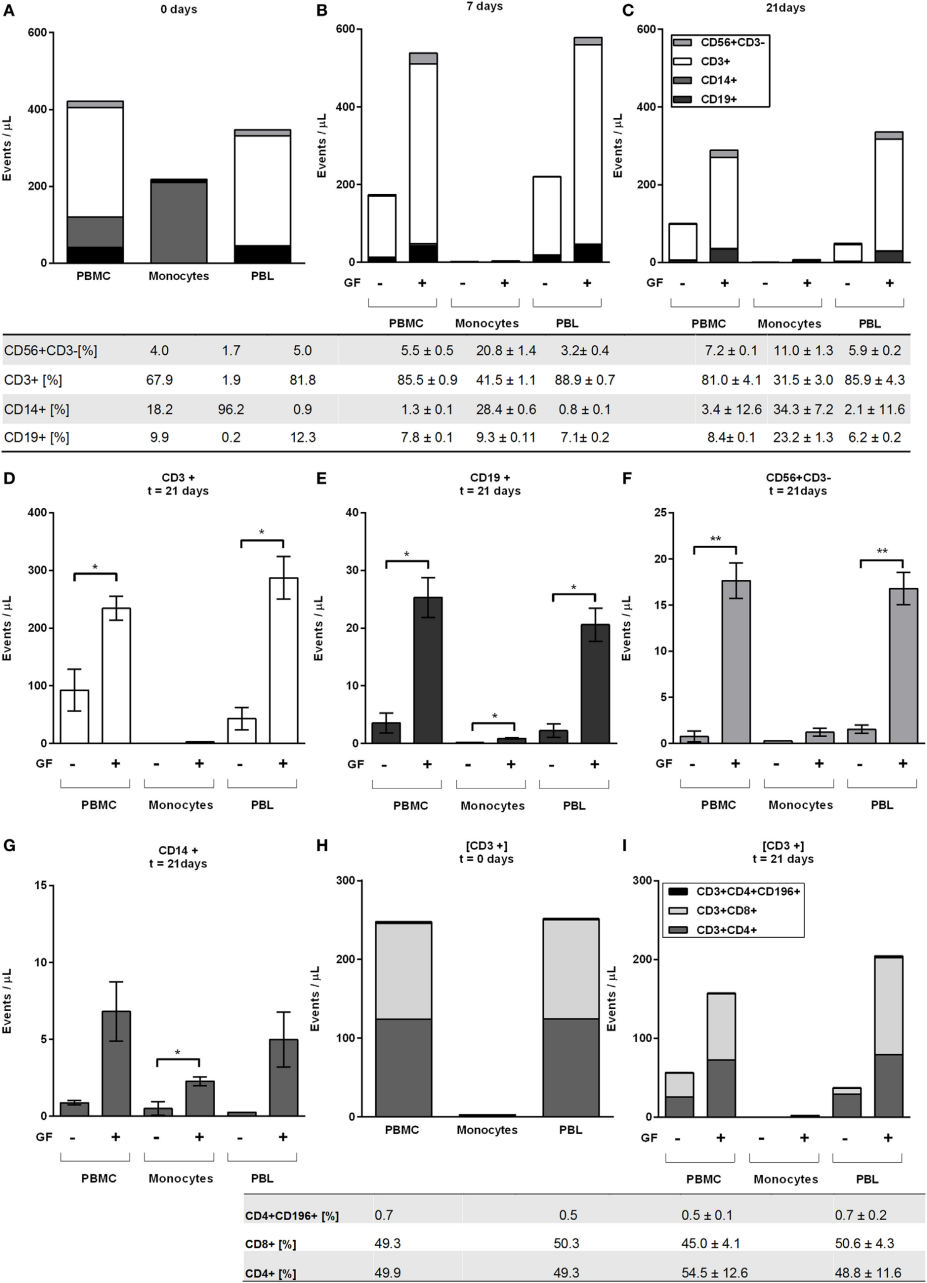
Peripheral blood lymphocytes cells survive in presence of gingival fibroblasts (GFs) after 21 days. Distribution of CD56+CD3−, CD3+, CD14+, and CD19+ as indicated by stack bars over time; **(A)** before coculturing (0 days), **(B)** after 7 days, and **(C)** after 21 days. The proportion of cells (in percentages ± SEM) in live populations of **(A)** monocultures and **(B,C)** cocultures are presented in the table below **(A–C)** per cell subtype for cocultures with GFs. **(D–G)** Specification of **(C)** at 21 days. **(D)** CD3+ T cells, **(E)** CD19+ B cells, **(F)** CD56+CD3− NK cells, **(G)** CD14+ monocytes. **(D–F)** For all peripheral blood lymphocytes (PBLs), significantly more events per microliters were detected in cocultures with peripheral blood mononuclear cells and PBLs. **(H)** Specification of CD3+ cell population at baseline levels where CD3+CD4+, CD3+CD8+, and CD3+CD4+CD196+ T cells are shown. **(I)** Specification of CD3+ cell populations after 21 days where CD3+CD4+, CD3+CD8+, and CD3+CD4+CD196+ T cells are shown. Tables below figures **(H,I)** represent proportion (percentages ± SEM) of cell populations per **(H)** monoculture and **(I)** coculture. Data are presented as events per microliter **(A–C,H,I)** ±SEM **(D–G)**. *n* = 10 GF donors, *n* = 2 buffy coats, *p < 0.05, **<0.01.

The immunohistochemical findings using CD3 and CD20 antibodies were confirmed with flow cytometry and extended for other leukocyte markers. At time points 0, 7, 14, and 21 days, flow cytometry was used to assess the number of monocytes (CD14+), T (CD3+), B (CD19+), and NK (CD56+CD3−) cells present in mono and cocultures of GFs (*n* = 10) with PBMCs, monocytes, and PBLs. Flow cytometric measurements are shown as events per microliter representing the actual counts per sample (Figure 4). The initial baseline cell distribution (Figure 4A) of monocultures shows a distribution of >96% monocyte purity with <4% lymphocytes in the CD14+ fraction and *vice versa* in the CD14− fraction. The PBMC fraction showed a baseline distribution of 18.2% CD14+, 67.9% CD3+, 4.0% CD56+CD3− and 9.8% CD19+ cells. After 7 days of culturing, only limited number of cells were observed in monocultures of PBMCs, monocytes, or PBLs without GFs (<220 events/μL) indicating that PBMCs rarely survive in the absence of GFs. In the presence of GFs, CD14+ cells were not detected after 7 days, which could be due to differentiation of monocytes toward osteoclasts, during which they may lose CD14 expression. In the presence of GFs, retention, and survival of PBLs is seen after 7 and 21 days (Figures 4B,C), which indicates a survival effect of GFs on PBL cells.

At 7 days, abundantly more CD3+ cells were present in cocultures (Figure 4B) than at baseline (Figure 4A): approximately, a quarter of T cells remained after 7 days in the absence of GFs, while in the presence of GFs, the T cells persisted (Figure 4B). Even after 21 days, the vast majority of persisting cells observed in both GF cocultures of PBMCs and PBLs were CD3+ T cells (81.0 ± 4.1, 85.9 ± 4.3, respectively, percentages ± SEM), which represented the dominant lymphocyte cell population in both cocultures (Figures 4C,D). For CD19+ B cells, a similar trend was observed. Cocultures of both PBMCs (*p* = 0.02) and PBLs (*p* = 0.02) with GFs contained significantly more CD19+ cells than monocultures (Figure 4E). Significantly more CD3+ T, CD19+ B, and CD56+ CD3− NK cells survive in PBMC and PBL cocultures with GFs (Figures 4D,G), indicating that GFs contribute to the survival of a broad range of lymphocytes. Overall, no difference between PBMC or PBL GF cocultures was observed, which indicates that monocytes do not play a role in the retention and survival of PBLs in the presence of GFs. No significant difference was seen in monocyte numbers in cocultures of PBMCs and PBLs, possibly indicating that minute contaminating numbers of CD14+ cells had a survival advantage in the presence of a multitude of other leukocytes. Significantly more CD14+ cells were present in monocyte cocultures than in monocultures (Figure 4G). Next to CD3+ T, CD19+ B, and CD56+CD3− NK cells, inflammatory responses in the oral cavity involve a mucosal efflux of neutrophils, which have migrated from the blood circulation. For this reason, we investigated the neutrophil–GF interaction (data not shown) and found no survival of neutrophils after 3 days.

Since the majority of cells were CD3+ T cells, two T-cell subsets were investigated with anti-CD4+, CD8+ for the detection of Th and Tc cells, respectively. In addition, one Th subset related to bone metabolism was investigated (20–22). Th17, known to be important in inflammation was detected with CD196+/CCR6 markers. At baseline (Figure 4H), an equal distribution of CD4+ and CD8+ was observed with a few (0.5–0.7%) CD3+CD4+CD196+ cells. Consistently, the same trend was observed for T subsets where more CD4+, CD8+, and CD196+ cells survived in the presence of GFs than without GFs after 21 days (Figure 4I). Remarkably, CD3+CD4+CD196+ cells remained solely in the presence of GFs (Figure 4I). Whether measured within PBMCs or PBLs cocultures, the distribution of CD3+ cell subsets CD4+ and CD8+ cells remained constant over time (Figure 4I).

### Retention and Survival of PBLs in Cocultures With GFs Over Time

**Figure 5:**
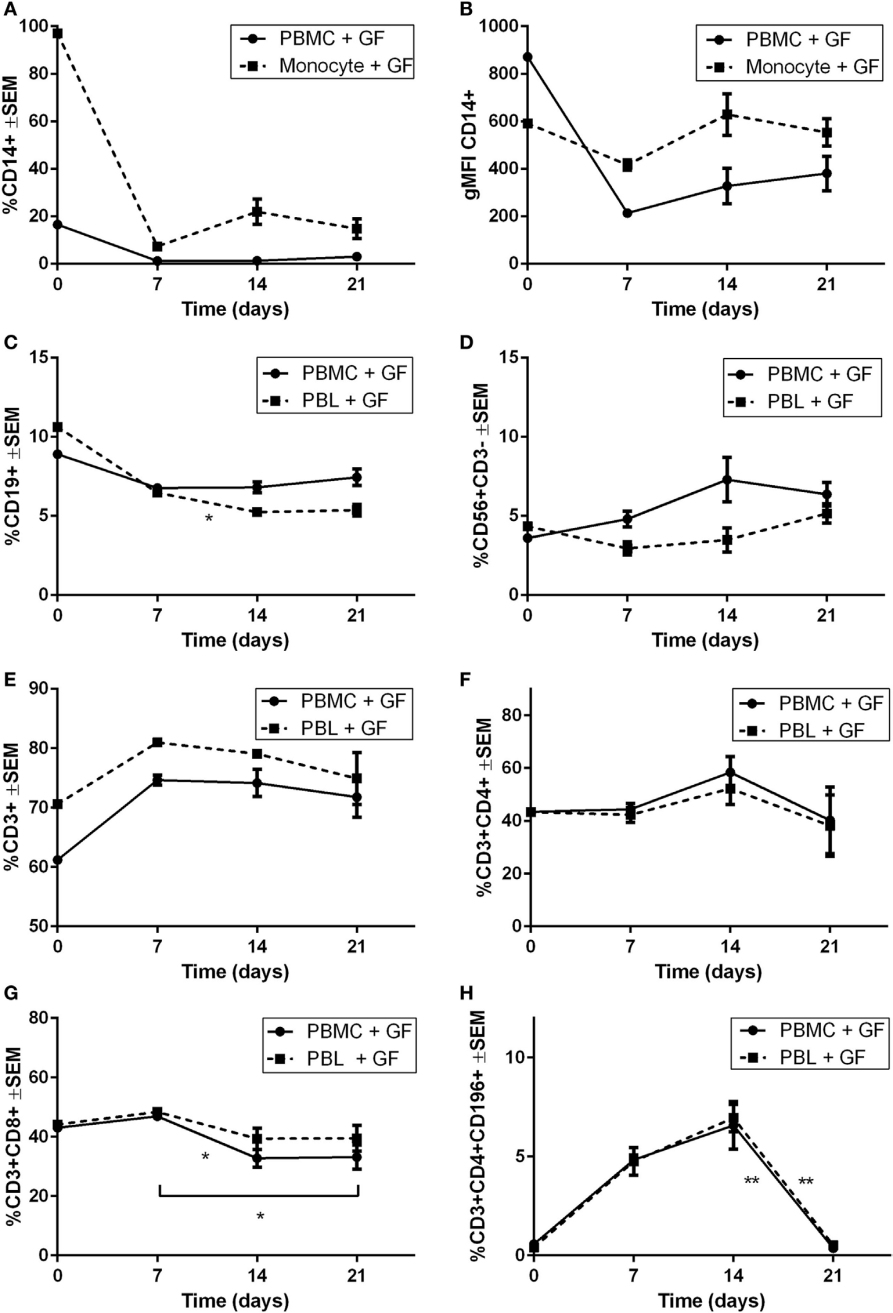
Survival and retention of peripheral blood lymphocytes in coculture of gingival fibroblast (GF) over time. Percentages of cells present in cocultures. **(A)** The proportion of CD14+ cells (percentage ± SEM) present in GF cocultures with peripheral blood mononuclear cells (PBMCs) (solid line) and monocytes (dotted line). **(B)** Geometric mean fluorescence intensity (gMFI) of CD14+ cells over time of GF cocultures with PBMCs (solid line) and monocytes (dotted line). No significant difference of CD14+ percentage **(A)** or gMFI **(B)** after 7 days was observed. Percentages ±SEM of **(C)** CD19+, **(D)** CD56+CD3−, **(E)** CD3+, **(F)** CD3+CD4+, **(G)** CD3+CD8+, **(H)** CD3+CD4+CD196+ cells present in GF cocultures with PBMCs (solid line) and peripheral blood lymphocytes (PBLs) (dotted line). No significant increase or decrease of percentage **(D)** CD56+CD3−, **(E)** CD3+, **(F)** CD3+CD4+ cells is shown over time in both cocultures of PBMCs and PBLs. **(C)** A significant decrease of CD19+ cells after 7 days is shown in PBL cocultures. Significant decrease of CD3+CD8+ cells is shown after 7 days in PBMC cocultures. A significant decrease of CD3+CD4+CD196+ cells after 14 days is shown in PBMC and PBL cocultures. Time point 0 represents monocultures of **(A,B)** monocytes, **(C–H)** PBMCs, and PBLs. All significant differences were tested after 7 days since time point 0 represents a characterization of one experiment. *n* = 10 GF donors, *n* = 2 buffy coats, *p < 0.05, **<0.01.

Heterogeneous cell populations of GF cocultures with PBMCs were characterized over time using flow cytometry. Since limited quantities of cells (<220 events/μL) were detected in monocultures of PBMCs, monocytes, or PBLs on plastic after 21 days, solely, the cocultures are shown. Flow cytometric measurements are shown as percentages of the live-gated populations. No PBLs were detected in cocultures with monocytes since initial baseline levels showed <4% PBLs. Therefore, the leukocyte constitution was no further analyzed in the CD14+ cocultures, explaining the exclusion of PBL coculture data for CD14+ proportions. Figure 5 displays the percentage of different cell subsets that were characterized over time. After 7 days, a steep decline in CD14+ cells was observed, which continued consistently for another 14 days (Figure 5A). Geometric mean fluorescence intensity (gMFI) (Figure 5B) was plotted for CD14+ referring to the fluorescence intensity of each event indicating the average expression of CD14+ per cell. Mean fluorescence intensities were stable, which indicates no difference in CD14 expression while taking into account the steep decrease of cell numbers indicating an increase of CD14+ per cell. A significant decrease of CD19+ cells was observed in PBL cocultures (*p* = 0.045), whereas no significant difference was observed in PBMC cocultures (Figure 5C). No significant changes over time were seen in CD56+CD3− (Figure 5D) and CD3+ (Figure 5E) populations, which did show a survival effect in the presence of GFs. An increase of >10% in both PBL and PBMC cocultures was seen at day 7 in CD3+ population. In the CD3+ population, Th subsets (CD3+CD4+, Figure 5F) showed no differences over time. After 7 days, a decrease of CD3+CD8+ T cells was observed in PBMC cocultures. Interestingly, a sharp increase of up to 5–7% of CD196+ Th17 cells was observed at 7 and 14 days, while thereafter, a significant (*p* = 0.0044, *p* = 0.0034) decline of CD196+ Th17 cells was seen between 14 and 21 days in PBMC and PBL cocultures, respectively (Figure 5H).

### LFA-1/Intercellular Adhesion Molecule 1 (ICAM) Expression Is Induced by GF–Lymphocyte Interactions

**Figure 6:**
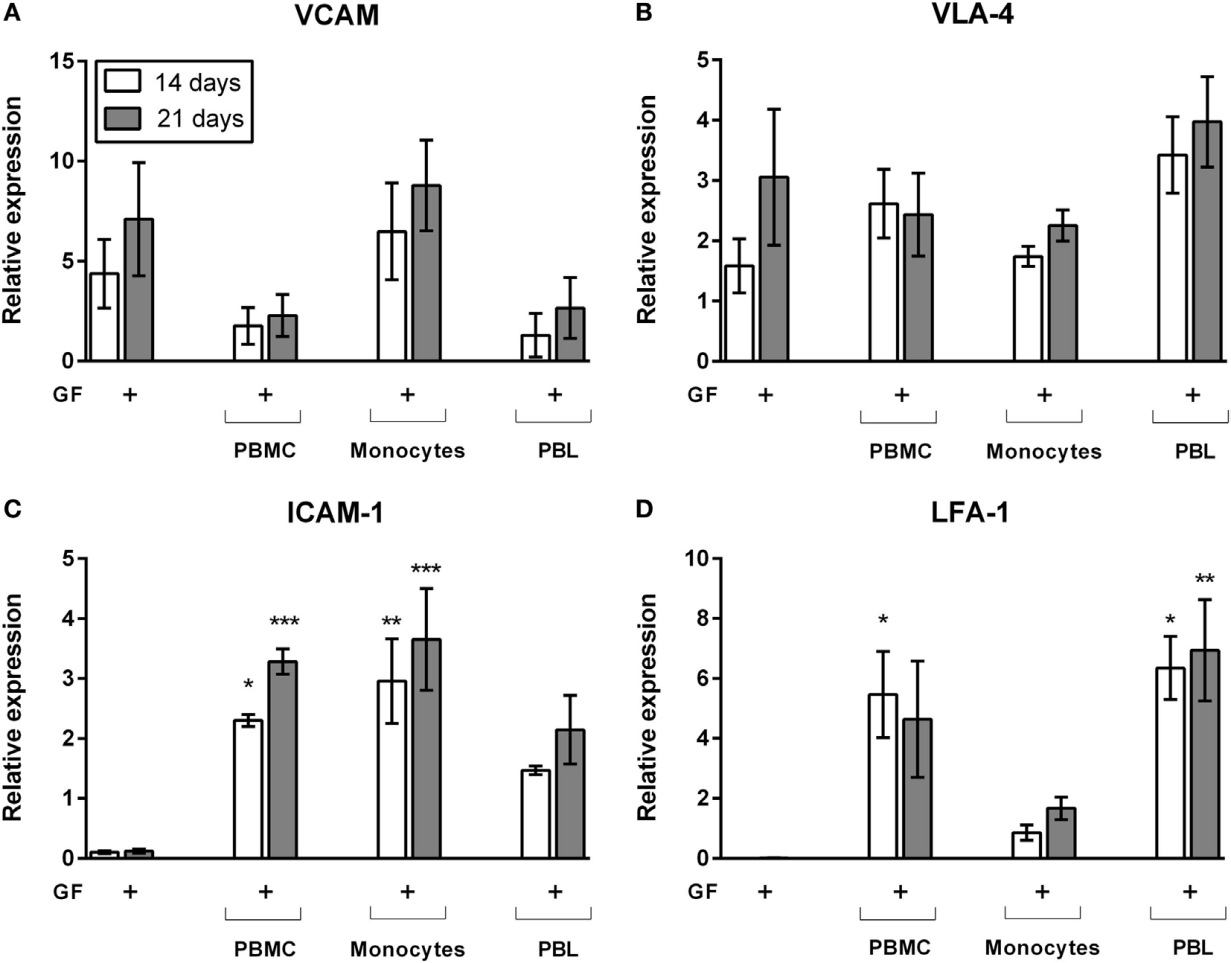
Gene expression of cell adhesion molecules of gingival fibroblast (GF) mono- and cocultures. Relative gene expression of adhesion molecules after 14 (white bars) and 21 days (gray bars) of culture. Gene expression of **(A)** VCAM, **(B)** very late antigen-4 (VLA-4), **(C)** intercellular adhesion molecule 1 (ICAM-1), and **(D)** LFA-1. No significant differences were found in VCAM and VLA-4 expression **(A,B)** while LFA-1 and ICAM-1 **(C,D)** expression is significantly increased in cocultures in comparison to GF monocultures. No significant differences were found over time. All genes are expressed relative to the geometric mean of housekeeping genes hypoxanthine phosphoribosyltransferase (HPRT) and porphobilinogen deaminase (PBGD). Data are presented as means ± SEM. All significant differences were compared to GF monocultures. *n* = 4 GF donors, *p < 0.05, **<0.01, ***<0.001.

Right from the start of culture, leukocytes adhered strongly to GFs. This was assessed by rigorously moving the plates in a horizontal position while observing the cells under the microscope. Cells remained attached to fibroblasts after these actions. Previously, we showed by interfering with lymphocyte-function-associated antigen-1 (LFA-1) blocking antibodies that T cells and monocytes use the LFA-1/ICAM-1 resulting in a strong inhibition of osteoclast formation (23). With qPCR, we identified possible key players for GF–leukocyte interactions leading to the retention of leukocytes in the presence of GFs. Since immunohistochemical stainings and flow cytometry results showed that limited numbers of cells survive on plastic after 14 and 21 days, only the gene expressions of GF cocultures was tested. Gene expression of GF monocultures and GF cocultures after 14 days (white bars) and 21 days (gray bars) are shown in Figure 6. Expression of vascular cell adhesion protein 1 (VCAM, Figure 6A) and very late antigen-4 (VLA-4, Figure 6B) were not modified by the presence of PBMCs, monocytes, or PBLs. This indicates that VCAM and VLA-4 expression is likely unaffected by, and thus not crucial for GF–lymphocyte interactions. ICAM-1 (Figure 6C) is significantly increased in PBMC cocultures. Monocytes appear to play an important role in ICAM-1 gene expression since PBL’s ICAM-1 expression was not significantly enhanced compared to GF monoculture (Figure 6C). LFA-1 expression was significantly increased in GF cocultures with PBMCs and PBLs (Figure 6D). Therefore, LFA-1 is likely the important lymphocyte adhesion molecule by which lymphocytes adhere to GFs. LFA-1 expression is significantly lower (*p* = 0.0054, *p* = 0.008 at *t* = 14, 21 days of culture, respectively) in cocultures with monocytes when compared to PBL cocultures. This indicates the importance of LFA-1 for GF–PBL cell-interactions for the survival and retention of PBLs in GF cocultures. Importantly, expression did not differ over time, which indicates that it is a process, which is initiated before 14 days and stays constant after 21 days, explaining the survival and retention of lymphocytes in GF cocultures.

### GF Cocultures With Lymphocytes Induce Elevated Gene Expression and Secrete TNF-α

**Figure 7:**
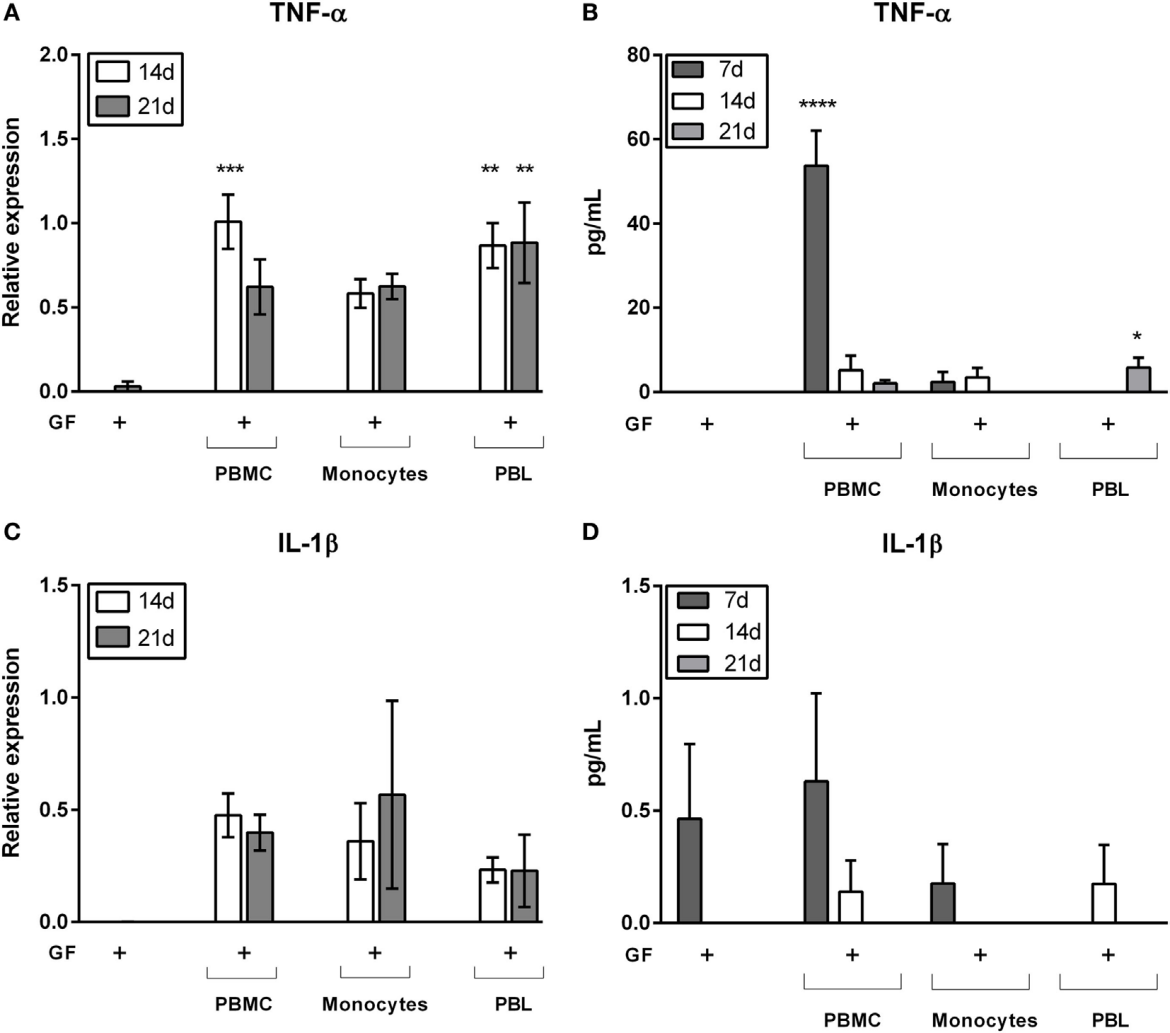
Gene expression and cytokine protein levels of tumor necrosis alpha (TNF-α) and interleukin 1 beta (IL-1β). Gene and protein expression of inflammatory cytokines **(A,B)** TNF-α and **(C,D)** IL-1β. Relative gene expression of inflammatory cytokines **(A)** TNF-α and **(C)** IL-1β after 14 (white bars) and 21 days (gray bars) of culture. Protein levels (pg/mL) of **(B)** TNF-α and **(D)** IL-1β after 7 (dark gray bars), 14 (white bars), and 21 days (gray bars). TNF-α **(A)** gene expression levels are significantly increased in gingival fibroblast (GF)-peripheral blood mononuclear cell (PBMC) cocultures (14 days) and GF-peripheral blood lymphocyte (PBL) cocultures (14 and 21 days) in comparison to GF monocultures. TNF-α **(B)** protein levels are significantly increased in GF-PBMC cocultures (7 days) and GF-PBL cocultures (21 days) in comparison to GF monocultures. No significant differences were found for IL-1β gene expression **(C)** and protein levels **(D)**. All genes are expressed relative to the geometric mean of housekeeping genes hypoxanthine phosphoribosyltransferase and porphobilinogen deaminase. Data are presented as means ± SEM. All significant differences were compared to GF monocultures. *n* = 6 GF donors for gene expression data, *n* = 5 GF donors for cytokine quantification data, *p < 0.05, **<0.01, ***<0.001, ****<0.0001.

Previously, we demonstrated that PBMC–PDL fibroblast interactions evoke an inflammatory response of IL-1β and TNF-α (24), the latter protein primarily expressed at 7 days of culture (25). In order to test the cell-type specific responses evoked by cocultures, now by PBMCs, monocytes, and PBLs, we next assessed gene expression and protein expression of GF mono- and cocultures with PBMCs, monocytes, and PBLs (Figure 7). Again, only the cocultures were tested since only few PBMCs, monocytes, and PBLs survived in absence of GFs (Figures 4B,C). Gene expression was assessed at 14 and 21 days and showed that expression of TNF-α was significantly increased in PBMC and PBL cocultures at 14 days and persisted up to 21 days in cocultures without monocytes (Figure 7A). When comparing the three coculture conditions, it became apparent that the presence of lymphocytes caused a higher gene expression of TNF-α. At the protein level, TNF-α (Figure 7B) was significantly increased in GF-PBMC cocultures (7 days) and GF-PBL cocultures (21 days) in comparison to GF monocultures. Interestingly, significantly higher TNF-α levels were quantified in PBMC cocultures after 7 days (53.7 ± 8.4 pg/mL, mean ± SEM), while this was not detected in monocyte and PBL cocultures. However, TNF-α levels significantly decreased after 14 days of coculture and were elevated solely in the PBL cocultures at 21 days. This indicates that a tripartite interaction of all cell subsets (monocytes, PBLs, and GFs) is needed for TNF-α expression at 7 days. Another important key inflammatory marker is IL-1β, which was analyzed for gene expression and protein secretion in GF mono- and cocultures. Again, IL-1β was only detected at the mRNA level in cocultures (Figure 7C). A similar non-significant pattern was observed in the secreted IL-1β protein levels (Figure 7D). Based on the strong TNF-α and non-significant IL-1β gene and protein expression profiles, we speculate that the TNF-α plays an important role in the lymphocyte survival and retention in presence of GFs.

### GFs Are Involved in the Proliferation of CD4+ and CD8+ T Cells

**Figure 8:**
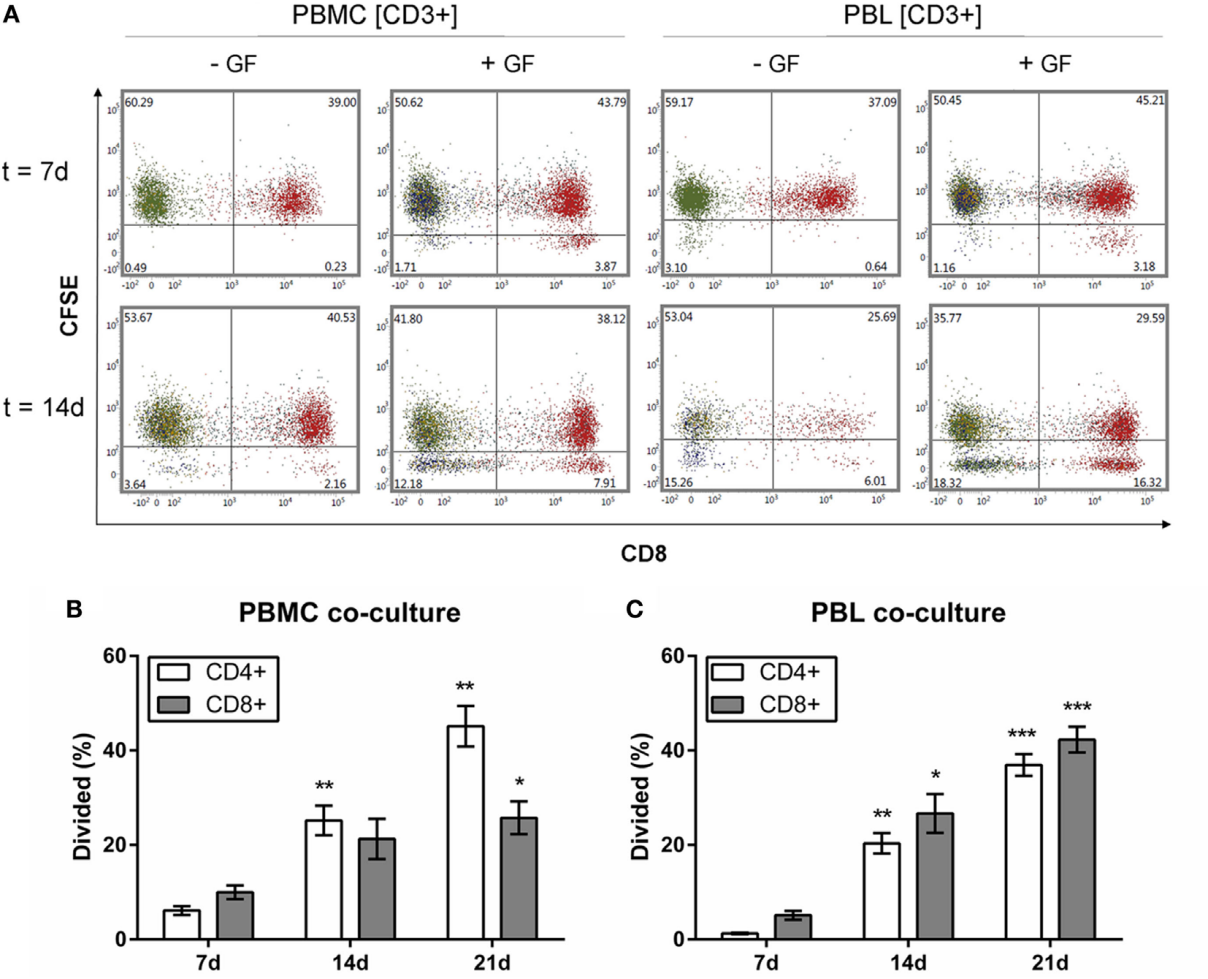
CD3+CD4+ and CD3+CD8+ cells proliferate in the presence of gingival fibroblasts (GFs). Quantification of proliferating Carboxyfluorescein succinimidyl ester (CFSE)-labeled CD3+ cells over time. **(A)** Dot plots of CD3+ cells within peripheral blood mononuclear cell (PBMC) (left panels) and peripheral blood lymphocyte (PBL) (right panels) cocultures after 7 (upper panels) and 14 days (lower panels). CFSE intensity is plotted on the *y*-axis and CD8+ is plotted on the *x*-axis. CD3+CD8+ cells are shown in red, CD3+CD8− (CD3+CD4+) cells are shown in green. Numbers in lower quadrants indicate the percentages of cells that have divided. **(B)** Quantification of the divided CD4+ (white bars) and CD8+ (gray bars) cells (normalized percentages ± SEM) in PBMC cocultures over time. **(C)** Quantification of the divided CD4+ (white bars) and CD8+ (gray bars) cells (normalized percentages ± SEM) in PBL cocultures over time. All significant differences were compared to 7 days. *n* = 5 GF donors, *p < 0.05, **<0.01, ***<0.001.

Gingival fibroblasts apparently played a crucial role in the PBL survival during 21 days of coculturing, showing a stable population of T, B, and NK cells. Over time, the majority of the retained population in GF cocultures was CD3+. Whether these retained CD3+ T cells and other retained leukocytes had the capacity to proliferate, was further investigated. Flow cytometric analysis was performed to determine the proliferation of (CFSE-labeled) PBMCs and PBLs after coculture with GFs over time. The intensity of CFSE decreases with every division indicating proliferation of cells after labeling. CFSE results for CD19+ B and CD56+ NK cells are not shown since no proliferation was observed for these subsets. Figure 8 shows the CFSE profiles of CFSE-labeled CD3+ cells within PBMC or PBL fractions cultured with or without GFs for 7 and 14 days. Based on initial gating (Figure 1E), CD3+CD8− cells are considered as CD3+CD8−CD4+ Th cells. PBMCs and PBLs cultured without GFs showed only minimal proliferation, while within cocultures, two separate populations of CD8−CD4+ and CD8+CD4− cells indicating division could be distinguished. A significantly higher percentage of dividing CD3+CD4+CD8− cells was seen after 7 days in both PBMC and PBL cocultures (white bars, Figures 8B,C). Significantly more CD3+CD8+ cells divided after 14 days in PBL cocultures (gray bars, Figure 8C), while this difference is only seen after 21 days in PBMC cocultures (gray bars, Figure 8B). The percentage of CD4+ and CD8+ T cells increased over time; from approximately 5% at 7 days, to approximately 20% at 14 days, to approximately 30–40% at 21 days. There was no significant difference in the percentage of dividing CD4+ and CD8+ cells. Also, no significant difference was observed between PBMC or PBL cocultures indicating that the presence of monocytes does not affect the survival or proliferation of CD3+ T cells. Both CD4+ and CD8+ cells proliferated, while previous results (Figures 4H,I and 5E) show a constant number of CD3+ cells over time. This indicates that the proliferate and cell death rates of CD3+ cells are in balance, resulting in a constant retaining CD3+ population. Finally, B cells and NK cells did not proliferate indicating that GFs specifically induced T cell proliferation.

## Discussion

In periodontitis, the persistence of a chronic inflammatory immune reaction stimulates the formation of osteoclasts that resorb alveolar bone. This chronic inflammation is characterized by a tissue infiltration of a heterogeneous effector cell population of the innate and adaptive immune response that interacts with resident cells of the periodontium. Until now, the exact mechanism of how chronic inflammation can lead to irreversible pathological bone resorption and which cells are responsible for this shift is unknown. Since egressing leukocytes encounter GFs after diapedesis, we hypothesized that GFs may play a role in the diversity of the cellular population at the site of inflammation. This study shows that GFs play a remarkable role in long-term retention, survival, and selective proliferation of PBLs, which is regulated by LFA-1/ICAM-1 expression and mediated by TNF-α cytokine production. Furthermore, we confirm previous findings that GFs stimulate osteoclastogenesis. These findings contribute to a better understanding of the onset of osteoclast formation, which is pathognomonic in periodontitis.

The formation of osteoclasts is regulated by several cytokines including receptor activator of nuclear factor kappa B (NF-κB) ligand [receptor activator of NF-κB ligand (RANKL)] and its decoy receptor osteoprotegerin (26). RANKL stimulates cell fusion of osteoclast’s monocyte precursors, osteoclast differentiation, and activation, leading to bone resorption. We observed that monocytes do not differentiate into osteoclasts in the absence of GFs. Thus, GFs can be posited to stimulate the differentiation of monocytes into osteoclasts. This is in line with previously reported data from our group (15) where significantly more (non-resorbing) osteoclasts were found in GF-PBMC cocultures than in PBMC monocultures. When compared to osteoclasts in cocultures with fibroblasts originating from PDL, GFs are relatively inhibitory (15). Similar inhibitory roles for GFs were described by Ujiie et al. who reported that GFs play an inhibitory role in osteoclastogenesis (27), possibly by secreting IL-4, which was shown to inhibit osteoclast formation. Here, we demonstrate that there was no difference in osteoclast formation between GF coculture conditions with either PBMCs or monocytes (*p* = 0.869) showing that unfractionated PBLs, although present, do not play a role in osteoclastogenesis in the presence of GFs. Within PBMCs, the monocytes are the only osteoclast precursor subset as previously demonstrated (13, 14, 28). After 7 days, a 90-fold 90% decrease of CD14+ was identified in GF-PBMC and GF-monocyte cocultures. One could speculate that the main population of monocytes underwent apoptosis due to a lack of supplemental essential growth factors in the culture media like macrophage colony-stimulating factor (M-CSF). Nevertheless, CD14+ expression was significantly higher after 21 days in the presence of GFs compared to monocultures of monocytes. Several studies (15, 29) have shown that M-CSF is produced by human GFs explaining the considerable number of living monocytes surviving in the presence of GFs that may have differentiated into TRACP+ mononuclear or MNCs. Thus, the remaining monocytes likely differentiated from CD14+ monocytes into CD14−/TRACP+ mononuclear osteoclast precursors, macrophages, and/or into CD14−/TRACP+ multinucleated osteoclasts. Survival and subsequent differentiation of monocytes into osteoclasts. Essential cytokines for the survival and subsequent differentiation of monocytes into osteoclasts are mainly provided by GFs and hardly by PBLs as there is no significant difference between osteoclast formation in PBMC and monocyte cocultures.

An interesting observation, which also demonstrates the role of GFs in osteoclastogenesis, is the retraction of GFs seen in cocultures either with monocytes or PBMCs. In contrast, no motility of GFs is seen in the absence of monocytes (thus pre-osteoclasts), showing that GF motility is dependent on the presence of monocytes and formed osteoclasts. Biologically, this retraction is relevant, since also *in vivo*, osteoclasts and osteoclast precursors have to migrate to the nearest bone surface, which can only be achieved when fibroblasts retract (15, 30). This retraction was also seen in another type of tooth-associated fibroblast: the PDL fibroblast. PDL fibroblasts are essential in the attachment of the tooth to the alveolar bone and recruitment of immune cells to the site of infection and have shown different responses in osteoclastogenesis and in gene expression to bacterial infections (15–18). Initial CD3+ and CD19+ stainings were performed in PDL fibroblast-PBMC cocultures (data not shown), indicating that the lymphocyte retention-function is not limited to GFs.

Remarkably, in our cocultures, the retaining TRACP-negative mononuclear cells interacted with TRACP+ cells. The number of interacting cells did not differ between the GF coculture and the monoculture (*p* = 0.0561). However, the *p*-value suggests that GFs somehow facilitate this interaction. Since the number of retaining PBLs in PBL/PBMC cocultures did not differ, it can be concluded that monocytes and osteoclasts do not play a role in the retention and survival of these PBLs and that GFs are mainly accountable for retention and survival. The retaining PBL population in GF cocultures of PBMCs comprised mainly of CD3, CD19/CD20, and CD56 cells. CD3+, exclusively expressed on the membrane of mature T cells, was found as the major population. In our culture system, with the exception of monocytes and polymorphonuclear granulocytes, GFs retain T, B, and NK cells constantly over time. Numbers of T (CD3+) and B cells (CD19+/CD20+) as well as NK cells (CD56+CD3−) were significantly increased in the presence of GFs as analyzed by immunohistochemistry and flow cytometry.

Since monocytes or osteoclasts are not responsible for the retention of PBLs, we could speculate on how GFs facilitate survival and retention of PBLs. Several studies (15, 27–30) demonstrated that fibroblasts are sources of pro- and anti-inflammatory cytokines, chemokines as well as cell adhesion molecules and orchestrate immune modulatory events for tissue homeostasis. For example, GFs produce interleukin-8 (IL-8) and TNF-α for recruitment of neutrophils (31). Interestingly, in our *in vitro* cocultures of GFs with neutrophils, the GFs did not play a role in the survival of neutrophils (data not shown). This was expected since the neutrophil is a short-living cell. Nevertheless, it is remarkable that GF apparently express the repertoire of crucial adhesion molecules and cytokines for the survival, proliferation of PBLs while this is not the case for neutrophils.

The paracrine production of pro-inflammatory cytokine IL-1 produced by GFs plays an important role in osteoclastogenesis, but also in the elevated expression of adhesion molecules in endothelial cells that anticipate the leukocyte migration from vascular lumen into interstitial tissue. Furthermore, it has been reported that GFs synthesize the pro-inflammatory cytokines IL-6 and tumor necrosis factor alpha (TNF-α), which activate the NF-κB cascade resulting in osteoclast formation and the pro-inflammatory cytokine IL-1, which stimulates bone resorption *via* prostaglandin E_2_ (32, 33). TNF-α and IL-1β cytokine production by GFs was analyzed in supernatants of GF mono- and cocultures with PBMCs, monocytes, and PBLs. Leukocyte retention was not mediated by IL-1β expression and secretion, but possibly by the production of TNF-α, which was only detected in cocultures. We confirm, with GFs, previous findings of Bloemen et al. (16) with PDL fibroblasts, who showed that TNF-α and other stimulators of osteoclast formation are expressed solely in cocultures. Monocyte–GF interactions were taken into consideration as an explanation for the role of GFs in osteoclastogenesis. Our findings corroborate the results of Domeij et al. (34) that monocytes induce ICAM-1 expression by GFs, which promotes monocyte survival, GF–monocyte interactions, and thus support osteoclastogenesis.

In our study, cell culture at early stages showed a firm attachment of PBLs to GFs as assessed after rigorously moving the cell culture plates in a horizontal position. Pro-inflammatory cytokines produced by GFs stimulate their binding with lymphocytes mediated by the LFA-I/ICAM pathway (9, 35). Adhesion of lymphocytes is also mediated by VLA-integrins and extracellular matrix receptors on GFs (35). VCAM and VLA-4 expression was indeed found in GF monocultures but was not increased after addition of PBMCs, monocytes, or PBLs indicating that they are not involved in monocyte survival, lymphocyte retention, and proliferation. Nevertheless, LFA-1 and ICAM-1 were only upregulated in cocultures supporting the essential presence of GFs for lymphocyte retention, survival, and proliferation.

With the current finding of PBL retention in GF cocultures, the role of retaining PBLs in osteoclastogenesis was questioned. The possible role of PBLs in the presence of GFs during osteoclastogenesis helps to understand how chronic inflammatory reactions may shift to pathological osteoclast formation in diseases like periodontitis and rheumatoid arthritis. Here, we would like to mention a restriction of the present study that the role of fibroblasts in the formation of osteoclasts was studied and not the osteoclastic activity *per se*. Likely, activators like RANKL (15, 36) have to be added to bring these formed osteoclasts to the state of resorbing osteoclasts. In periodontitis patients, a significantly greater number of inflammatory cells and osteoclasts are present at the site of inflammation (6). The human inflamed periodontal immune-cell network is characterized by subsets of T and B cells as well as neutrophils, in which the T cell subset was 10 times higher than in healthy individuals (37) suggesting their role in osteoclast formation and activity. Interestingly, more B and T cells could induce osteoclastogenesis since soluble RANKL cleaved from activated lymphocytes by a TNF-α converting enzyme contributes to osteoclastogenesis in periodontitis (38–41). This has been confirmed by other studies also showing that activated T cells (42–45) could positively or negatively contribute to osteoclast formation *in vitro*. Notably, T cells were the major population detected in GF cocultures with PBLs after 21 days. Our findings suggest that, if the PBLs contributed to osteoclast formation, the positive and negative contributions were in balance since monocyte and PBMC cocultured with GFs gave rise to similar numbers of osteoclasts. Nevertheless, it has been shown in mice that activated CD4+ T cells promote, while CD8+ T cells suppress osteoclastogenesis (41, 42). Choi et al. (42) also showed that cell–cell contact of osteoclast precursors and activated T cells is critical for osteoclastogenesis. Here, we show that the CD4+ T cells were found as the major T cell subset after 14 days suggesting an important role for resident CD4+ T cells in homeostasis and immunopathology of the gingiva. Overall, an equal distribution of CD4+/CD8+ subsets was found over time, likely nullifying a possible effect of T-cells on osteoclast formation.

Additionally, we found that the number of Th17 (CD3+CD4+CD196+) subset rises (7 days) and declines (14 days). This appears to correlate with what happens during a periodontal infection (5, 46, 47), where the population of Th17 is increased after approximately 1 week followed by a decline Activated CD4+ T cells are main sources for IL-17, which is mainly produced by the CD3+CD4+CD196+/CCR6 Th17 subset (44, 46, 47). Notably, IL-17 producing cells have been found to be an important player in the pathogenesis and onset of periodontitis (21, 48, 49). IL-17, when cocultured with mouse hematopoietic cells and primary osteoblasts induced MNCs (50). Multiple criteria of osteoclasts, like TRACP activity, calcitonin receptors, and increased pit formation were analyzed, confirming the finding. Additionally, Yago and colleagues (22) showed a mechanism by which IL-17 induces osteoclastogenesis from human monocytes alone, which was later on assigned to be mainly by intermediate monocytes (51). The temporal increase of Th17 cells observed in the present study may contribute to the differentiation of osteoclasts.

Other than a neutral retention and survival of PBLs in GF cocultures, it could be speculated that PBLs, especially T cells, proliferate in the presence of stimulatory cytokines. An increase of T cells of approximately 10 percent is observed after 7 days of coculture, while a decrease of T cells is observed without GFs. In the present study, the proliferation of PBMCs and PBLs was tracked with CFSE. The CFSE signal of both CD4+ and CD8+ cells decreased over time, indicative of proliferation while percentages of CD8+ cells significantly decrease after 7 days. Note that not all CD4+ and CD8+ cells proliferated, showing heterogenic responsiveness to GF. Clearly, proliferation did not appear in the absence of GFs. Also, no proliferation was seen in other leukocytes. Additionally, there is no significant difference between CD4+ and CD8+ cells indicating that GFs provide the essential survival and proliferative cytokines for both CD4+ and CD8+ cells since these do not differ in proliferative capacity in presence of GFs. Despite the demonstrated proliferation, the numbers of CD3+ cells remained constant over time, suggesting that probably a proportion of CD3+ went into apoptosis.

Our findings show that GFs support the retention and survival of T, B, and NK cells by LFA-1 expression. Additionally, TNF-α expression was only observed in the GF-PBMC cultures, indicating that the tripartite presence of GFs, monocytes, and lymphocytes is required for such an induction. Finally, we show that GFs stimulate the proliferation and differentiation of CD3+ cells, probably by expression of essential growth factors. Within the context of a periodontal inflammation, GF may thus contribute to both the temporization and the clearance of the inflammation by supporting the long-term survival of lymphocytes. On the other hand, retention and proliferation of lymphocytes mediated by GFs could lead to an accumulation of lymphocytes participating in local chronic inflammatory reactions, worsening the disease. This could be an explanation of the local accumulation of immune cells in gingival tissues, which were histopathologically examined by Dutzan et al. (37) and Thorbert-Mros et al. (6). A better understanding of osteoimmunological processes in which tooth-associated fibroblasts interact with immune cells could lead to a better understanding of the desired homeostasis to maintain periodontal health, while the current study has shown that GF–lymphocyte interactions are essential for osteoclastogenesis that is pathognomonic for periodontitis.

## Ethics Statement

The current study was exempt from ethical approval since Dutch law at time of tissue collection did not require ethical approval for human surgical waste material such as wisdom teeth. GFs were retrieved from healthy donors who agreed to donate the biological waste material (third molars with attached gingiva) after extraction of their wisdom teeth as part of their treatment in the Academic Center for Dentistry Amsterdam (ACTA) and stored in liquid nitrogen. Informed consent was obtained from all individuals and samples were coded to guarantee the anonymity of the donors as required by Dutch law. Researchers handling the fibroblasts (Carolyn G. J. Moonen, Sven T. Alders, Ton Schoenmaker, and Teun J. de Vries) could not retrieve the identity of the donors.

## Author Contributions

CM and TdV contributed to the conception and design of the study and conducted cytokine quantification experiments. SA performed immunohistochemical experiments. CM and HB designed and performed flow cytometry experiments. TS designed primers for gene expression experiments. CM and TS performed osteoclastogenesis and gene expression experiments. CM performed statistical data analyses and wrote the first versions of the manuscript, supervised by TdV. CM, TdV, SA, HB, TS, BL, EN, and TV contributed to manuscript revisions, read and approved the submitted version.

## Conflict of Interest Statement

The authors declare that the research was conducted in the absence of any commercial or financial relationships that could be construed as a potential conflict of interest.

## Abbreviations

BSA, bovine serum albumin; CD, cluster of differentiation; CFSE, Carboxyfluorescein succinimidyl ester; DAPI, 4′,6-diamidino-2-fenylindool; DMEM, Dulbecco’s minimal essential medium; FCS, fetal calf serum; GF, gingival fibroblast; gMFI, geometric mean fluorescence intensity; HPRT, hypoxanthine phosphoribosyltransferase; ICAM, intercellular adhesion molecule 1; IL-1β, interleukin 1 beta; LFA-1, lymphocyte-function-associated antigen-1; mAb, monoclonal antibodies; M-CSF, macrophage colony-stimulating factor; MNC, multinucleated cell; NFκB, nuclear factor kappa B; OC, osteoclast; PBGD, porphobilinogen deaminase; PBL, peripheral blood lymphocyte; PBMC, peripheral blood mononuclear cell; PDL, periodontal ligament; RANKL, receptor activator of NF-κB ligand; Tc, cytotoxic T cell; Th, T helper cell; TNF-α, tumor necrosis alpha; TRACP, tartrate resistant acid phosphatase; VLA-4, very late antigen-4.
